# Supplementary material for: A Confirmatory, Dual Center Nonhuman Primate Study on the Efficacy of Novel Oropharyngeal Spray Immunization With an Adenoviral Vector Vaccine Against RSV—Important Lessons Learned
Source: J Med Primatol. 2026 Oct 20;55(6):e70121. doi: 10.1111/jmp.70121 (PMC13682502; doi:10.1111/jmp.70121)
Supplement: Supplementary file 1 [file JMP-55-e70121-s001.pdf]

Supplementary Figure 1

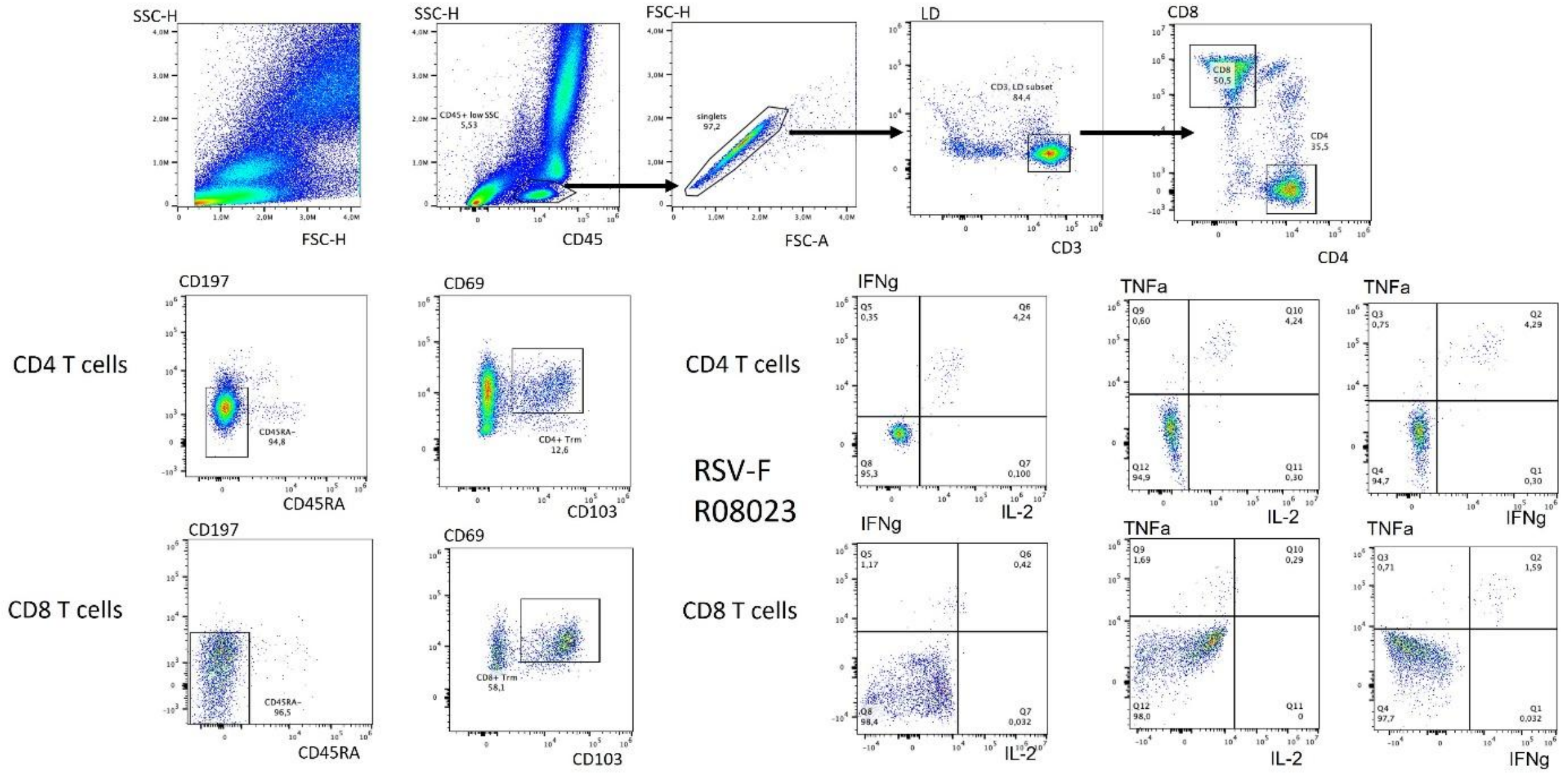

Suppl.Fig.1: Gating strategy for intracellular cytokine staining of BAL cells
Representative dot plots are shown for one BAL sample from the RSV vaccine group at BPRC (animal: R08023) which was stimulated with the FSV-F specific peptide pool.

Supplementary Figure 2

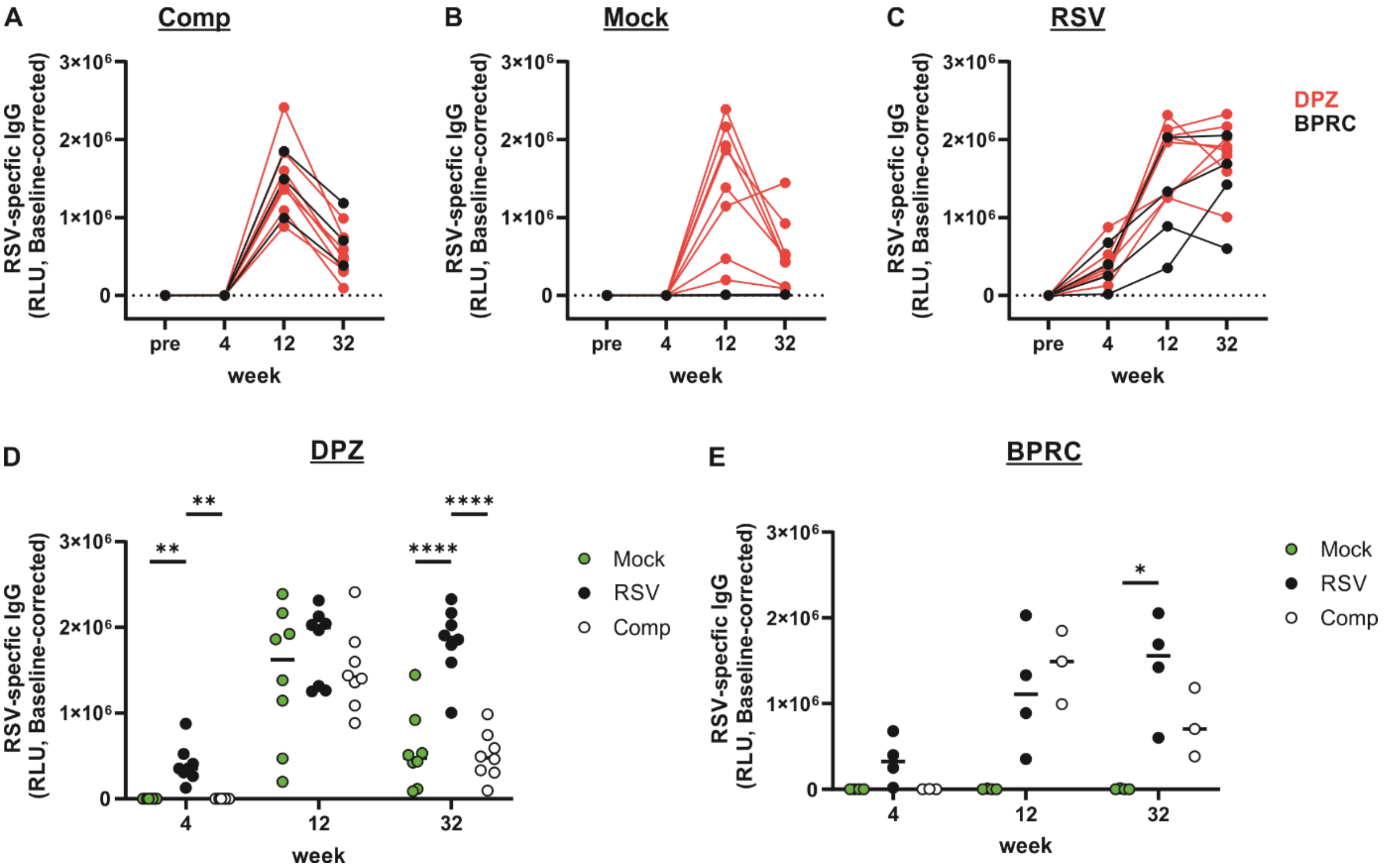

Suppl.Fig.2: RSV-specific antibody responses

Animals were immunized as illustrated in Fig.1. Sera were collected at indicated time points and diluted 1:500. RSV-specific IgG antibodies were determined by an ELISA using inactivated RSV particles as coating agent. The amount of IgG in serum is shown as RLU. To account for variance in the background binding of individual monkey sera, we subtracted the RLU values of the pre sera from all other samples for each individual animal. The time course of RSV-specific IgG levels is shown for each individual animal in the comparator (A), Mock-treated (B) or RSV-immunized (C) group. All animals from the DPZ are indicated by red symbols and lines, while the BPRC animals are marked in black. (A-C). The group comparison for each time point is shown for the DPZ (D) and the BPRC (E). Statistical significances were analyzed by two-way ANOVA followed by Tukey´s multiple comparisons test and p values indicate significant differences (*p < 0.05; **p < 0.01; ****p < 0.001). (n=8 for DPZ; n=3 for BPRC, one animal from the BPRC is excluded from the analyses due to a missing pre-treatment serum)

Supplementary Figure 3

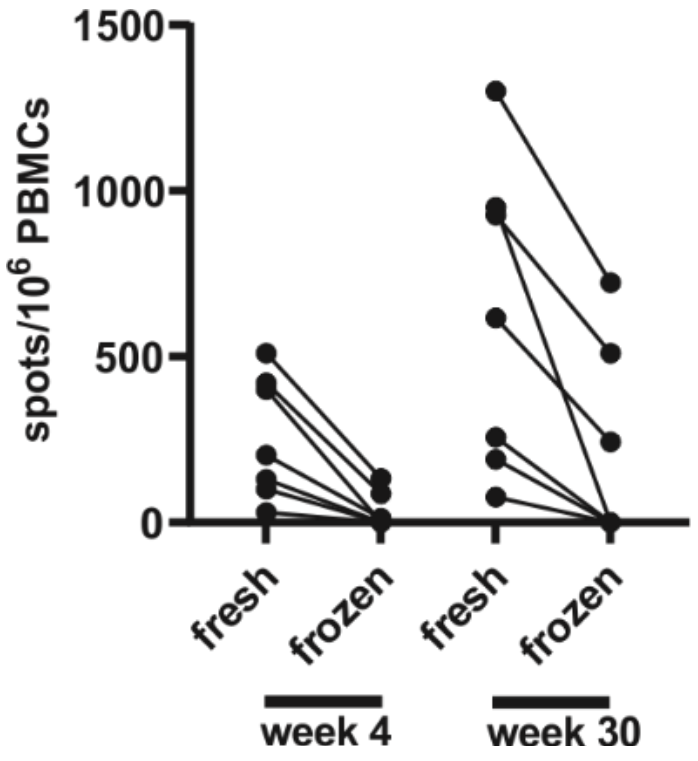

Suppl.Fig.3: Comparison of ELISPOT analyses between fresh and frozen PBMC samples
PBMCs were isolated at the indicated time points and either frozen and stored at -80° until the ELISPOT assays were performed or were directly restimulated for the ELISPOT assay. Values of samples from the RSV-immunized animals from DPZ are shown for the two time points (W4 and W30). Pairing of the individual values is indicated by the lines.
